# Physico-Chemical Properties of Granular Sorbents Based on Natural Bentonite Modified by Polyhydroxocations of Aluminum and Iron (III) by Co-Precipitation

**DOI:** 10.3390/molecules30010195

**Published:** 2025-01-06

**Authors:** Bakytgul Kussainova, Gaukhar Tazhkenova, Ivan Kazarinov, Marina Burashnikova, Raigul Ramazanova, Yelena Ivashchenko, Bekzat Saurbayeva, Batima Tantybayeva, Ainur Seitkan, Gulsim Matniyazova, Khalipa Sadiyeva, Aisha Nurlybayeva, Aidana Bazarkhankyzy

**Affiliations:** 1 Department of Chemistry, Faculty of Natural Sciences, L.N. Gumilyov Eurasian National University, 010000 Astana, Kazakhstan; nurbol-bakytgul@mail.ru; 2 Department of Physical Chemistry, Saratov State University, 410000 Saratov, Russia; kazarinovia@mail.ru (I.K.); burashnikova_mm@mail.ru (M.B.); 3 School of International Engineering, D. Serikbayev East Kazakhstan Technical University, 070004 Ust-Kamenogorsk, Kazakhstan; raigul_77_33@mail.ru (R.R.); elena.ivashchenko06@gmail.com (Y.I.); saurbaeva71@mail.ru (B.S.); 4 Department of Chemistry, Higher School of Natural Sciences, Sarsen Amanzholov East Kazakhstan State University, 070000 Ust-Kamenogorsk, Kazakhstan; bati_54@mail.ru; 5 Higher School of Natural Sciences, Astana International University, 010000 Astana, Kazakhstan; seitkanainur.77@mail.ru (A.S.); gulsim.matniyazova@mail.ru (G.M.); 6 Department of Chemistry and Chemical Technology, Faculty of Technology, M.Kh. Dulaty Taraz Regional University, 080000 Taraz, Kazakhstan; xalipa71@mail.ru; 7 Department of General and Biological Chemistry, Astana Medical University, 010000 Astana, Kazakhstan; bazarkhankyzy.a@gmail.com; 8 Republican Collection of Microorganisms, 010000 Astana, Kazakhstan

**Keywords:** bentonite, sorbent, sorption of bichromate, arsenate anions, nickel cations, specific surface area, polyhydroxocations, co-precipitation

## Abstract

The physicochemical and adsorption properties of granular sorbents based on natural bentonite and modified sorbents based on it have been studied. It was found that modification of natural bentonite with iron (III) polyhydroxocations (mod. 1_Fe_5 GA) and aluminum (III) (mod. 1_Al_5 GA) by the “co-precipitation” method leads to a change in their chemical composition, structure, and sorption properties. It is shown that modified sorbents based on natural bentonite are finely porous (nanostructured) objects with a predominance of pores measuring 1.5–8.0 nm, with a specific surface area of 55–65 m^2^/g. Modification of bentonite with iron (III) and aluminum compounds by the “co-precipitation” method also leads to an increase in the sorption capacity of the obtained sorbents with respect to bichromate and arsenate anions and nickel cations by 5-10 times compared with natural bentonite. The obtained sorption isotherms were classified as Langmuir type isotherms. Kinetic analysis showed that at the initial stage the sorption process is controlled by an external diffusion factor, i.e. refers to the diffusion of sorbent from solution into a liquid film on the surface of the sorbent. Then the sorption process begins to proceed in a mixed diffusion mode, when it limits both the external diffusion factor and the internal diffusion factor (the diffusion of the sorbent to the active centers through the system of pores and capillaries). To determine the contribution of the chemical stage to the rate of adsorption of bichromate and arsenate anions and nickel(II) cations with the studied granular sorbents, kinetic curves were processed using the equations of chemical kinetics (pseudo-second-order model). As a result, it was found that the adsorption of the studied anions by modified sorbents based on natural bentonite is best described by a pseudo-second-order kinetic model. It is shown that the use of natural bentonite for the development of technology for the production of granular sorbents based on it has an undeniable advantage, firstly, in terms of its chemical and structural properties, it is easily and effectively modified, and secondly, having astringent properties, granules are easily made on its basis, which turn into ceramics during high-temperature firing. The result is a granular sorbent with high physical and mechanical properties. Since bentonite is an environmentally friendly product, the technology of recycling spent sorbents is also greatly simplified.

## 1. Introduction

The pollution of reservoirs with heavy metals and toxic anions poses [1] a significant threat to ecosystems and human health [2,3]. As industrial production has increased, so has the volume of wastewater containing heavy metal ions and various salts, including toxic compounds such as arsenic [4,5], chromium [6], and selenium [7], primarily from anthropogenic sources. Unlike natural carbonate and sulfate [8] ions, these heavy metal ions are hazardous due to their toxicity and potential for bioaccumulation [9]. To effectively remove such pollutants, adsorption purification methods are widely employed, as they can target a broad range of contaminants in water bodies [10].

Bentonite clays have garnered considerable attention as sorbents due to their unique properties and high efficiency in removing heavy metals [11,12,13]. Bentonite, a member of the smectite clay group [14], is an aluminosilicate characterized by a high surface area and cation exchange capacity, making it particularly effective for adsorption and ion exchange [15,16]. Its layered structure enhances its adsorption capabilities, allowing for the efficient capture of heavy metal cations. Furthermore, bentonite can be modified and granulated to improve its sorption properties and stability under varying operational conditions [17].

One well-established method for enhancing the adsorption capacity of clays is the modification of their structure by introducing metal polyhydroxocations [18]. Complexes formed with metals such as aluminum and iron strengthen interlayer bonds [15], resulting in improved mechanical resistance, thermal stability, and adsorption capacity. This approach enables modified clay minerals to find applications across diverse fields, including water purification, polymer nanocomposite [19] development, and geotechnical applications.

In our previous research, we presented results on the adsorption of bichromate and arsenate anions using powdered sorbents based on bentonite [20,21]. However, this powdered form has exhibited limitations in durability and mechanical stability. In the current study, we employed the same methods to produce granular sorbents, sized 1–2 mm. Our findings indicate that granulated sorbents derived from bentonite demonstrate superior adsorption properties compared to their powdered counterparts. The granules’ structure and larger size (1–2 mm) provide a greater contact area with pollutants and enhance resistance to mechanical influences. Unlike powdered forms, granules maintain their structural integrity during repeated use, enabling them to sustain high adsorption efficiency over extended operational cycles.

Additionally, granulation addresses the critical issue of peptization (dispersion) of small sorbent particles in aqueous environments, a common problem with powdered materials [22,23]. Dispersion can lead to sorbent loss and decreased water purification quality. In contrast, granular sorbents do not disperse in water, making them easier to use in industrial filters and columns while minimizing maintenance requirements and material losses [24,25]. These advantages render granular sorbents more suitable for industrial treatment systems, where stability, reliability, and the potential for repeated use are essential [26].

The objective of this work is to develop granular sorbents based on bentonite from the Pogodayevo deposit (Republic of Kazakhstan), modified with aluminum and iron (III) polyhydroxocations using the “co-precipitation” method. This modification aims to enhance the adsorption properties of the sorbents concerning nickel (II) cations and arsenate and bichromate anions.

## 2. Results and Discussion

### 2.1. Characteization of the Adsorbent

#### 2.1.1. Elemental Composition of the Studied Sorbents

The chemical and mineral compositions of granulated modified sorbents based on bentonite are presented. Table 1 displays the elemental compositions of the studied sorbents, which were modified with polyhydroxocations of iron (III) and aluminum (III) using the “co-precipitation” method. The quantitative analysis of the elemental composition was conducted using an energy-dispersive X-ray fluorescence spectrometer (EDX-720, Shimadzu, Kyoto, Japan) with standard parameters:

- Natural bentonite of the Pogodayevo deposit—mod. 1 GA (granulated annealed);
- Natural bentonite (mod. 1) modified with iron (III) polyhydroxocations by the “co-precipitation” method (5 mmol [Fe^3+^]/g of bentonite)—mod. 1_Fe_5-c GA;
- Natural bentonite (mod. 1) modified with polyhydroxocations of aluminum (III) by the “co-precipitation” method (5 mmol [Al^3+^]/g of bentonite): mod. 1_Al_5-c GA.

**Table 1:** Elemental composition of the studied bentonite-based sorbents.

Samples of Sorbents	The Content of the Element, wt. %						
	Al	Fe	Si	Ca	K	Ni	Ti
mod. 1 GA	7	48	17	4	12	10	2
mod. 1_Al_5-c GA	19	47	13	2	10	7	2
mod. 1_Fe_5-c GA	6	70	9	2	5	6	2

**Figure 1:**
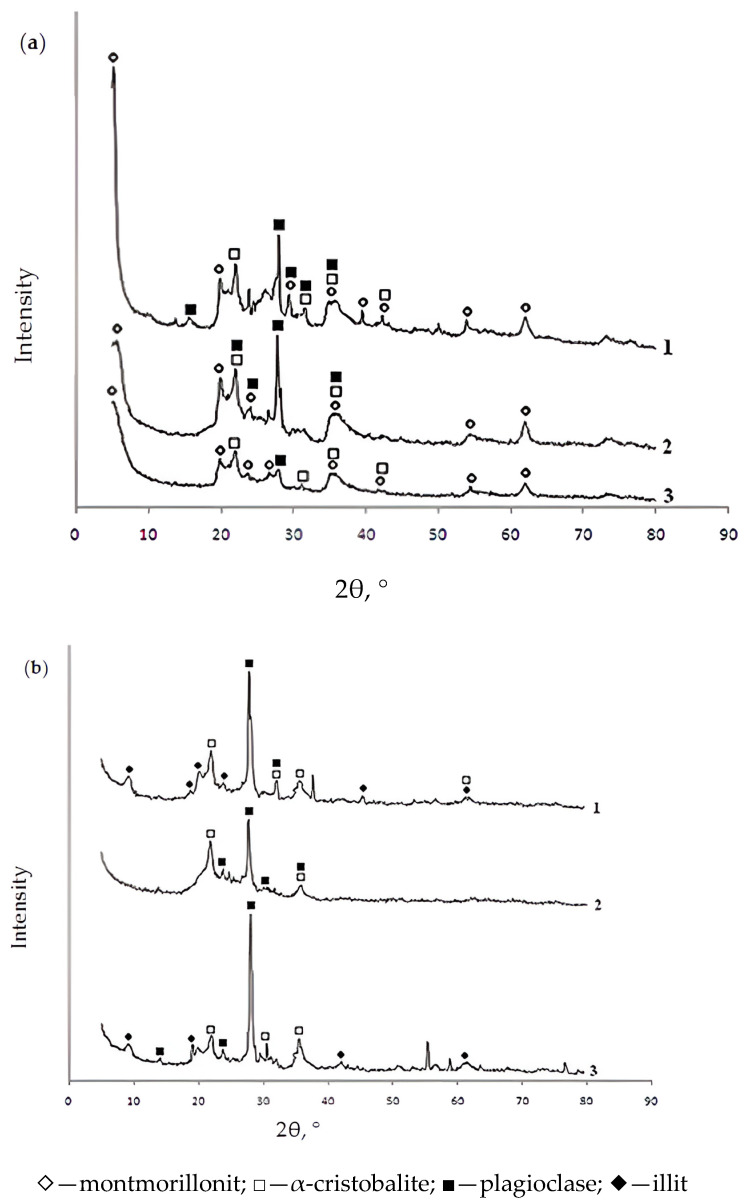
X-ray diffractograms of powdered (**a**) and granular (**b**) samples of the studied sorbents based on bentonite from the Pogodayevo deposit (Kazakhstan) annealed in an argon atmosphere at 500 °C: 1—mod 1; 2—mod 1_Al_5-c; 3—mod 1_Fe_5-c.

Table 1 confirms that sorbents derived from natural bentonite are aluminosilicates. An increase in the concentration of the modifying component led to a higher concentration of the corresponding element in the bentonite sample. This change is due to the replacement of bentonite cations, particularly calcium cations. The magnesium and sodium content in the modified bentonite samples and sorbents could not be determined because the measurement range of the dissipated energy was insufficient. Since the EDX-720 (Shimadzu, Kyoto, Japan) X-ray fluorescence spectrometer operates within the Na-U range, it is unable to detect the minimal amounts of light metals present in the samples, which are instead recorded as background noise. Figure 1 shows the X-ray diffractograms of the initial bentonites and the modified sorbents based on them. XRD analysis was performed on a DRON-8T diffractometer (Bourevestnik, Saint Petersburg, Russia) using an X-ray tube with a copper anode (Cu-Kα radiation).

As follows from the X-ray diffractograms, the additional introduction of aluminum and iron (III) polyhydroxocations into bentonite by the “co-precipitation” method does not result in a change in the mineral and phase composition of bentonite (in all considered cases, the observed minerals are montmorillonite, α-cristobalite, and plagioclase). Annealing of the studied samples at 550 °C in an argon atmosphere leads to changes in the mineral composition of the sorbents. In the initial bentonite and in samples modified with iron (III) polyhydroxocations, the mineral illite appears in place of montmorillonite.

#### 2.1.2. The Porous Structure of the Studied Sorbents

The results of the study of the porous structure of natural bentonites and their compounds modified with polyhydroxocations of iron (III) and aluminum (III) by the “co-precipitation” method are presented in Table 2. The tabular data show that the modification of bentonites leads to an increase in the number of micro- and mesopores and a decrease in the number of macropores compared with the original bentonites. Most of the pores in all modified samples are in the size range of 1.5–8.0 nm.

This redistribution of pore sizes also resulted in a significant increase in the specific surface area of the modified sorbents. The modification of bentonite with iron (III) and aluminum polyhydroxocations increased the specific surface area of the sorbent samples to 54 and 65 m^2^/g, respectively.

#### 2.1.3. Granulometric Composition of the Studied Powdered Sorbents

Granulometric analysis was carried out to obtain information on the particle size distribution in powdered samples of the sorbents under study. The particle size distribution was determined using a laser diffraction particle size analyzer, SALD-2201, manufactured by SHIMADZU (Kyoto, Japan) with a “dry” prefix, by laser diffraction. The principle of operation of this method is based on the registration of optical radiation, which is scattered by particles in the analyzer cuvette. A highly sensitive multi-element detector captures the laser radiation scattered by the particles. The particle size distribution is calculated based on the measured dependence of the scattered radiation intensity on the scattering angle. The measurement results are presented in Table 3.

It can be seen from the data in Table 3 that particles ranging in size from 0.5 to 500 microns are present in all samples. However, most of the particles are between 50 and 200 microns in size. According to the classification presented in [22], these samples are classified as coarse-dispersed. The presence of larger fractions in the samples and an underestimated content of smaller ones may be associated with particle aggregation. It should be noted that the particle size significantly exceeds the pore size for all samples; therefore, all sorbents exhibit high “internal porosity”.

#### 2.1.4. Morphology of the Studied Powdered Sorbents

**Figure 2:**
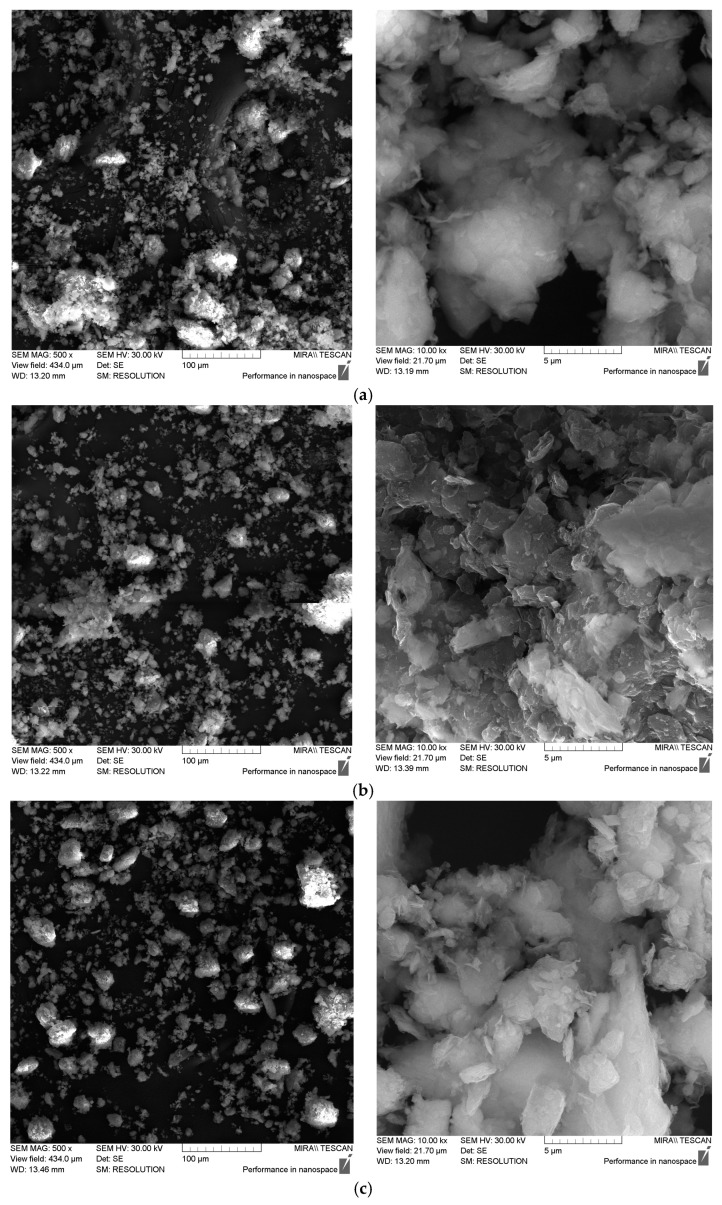
SEM photographs of the studied samples of powdered sorbents. (**a**) mod. 1 GA, (**b**) mod. 1_Al_5-c GA, (**c**) mod. 1_Fe_5-c GA.

Figure 2 shows photographs of the powdered sorbent samples studied, obtained using a scanning electron microscope (SEM) at various magnifications.

The analysis of the SEM images of the studied powdered sorbents showed that they are polydisperse systems. Modification of bentonite with polyhydroxocations of aluminum and iron (III) leads to a decrease in particle aggregation and, consequently, an increase in the specific surface area of the modified sorbents. This is especially evident for aluminum-modified sorbents (sample mod. 1_Al_5_c GA). However, as shown in Table 2, the increase in the specific surface area of modified sorbents is more closely associated with a change in their internal porosity, specifically with an increase in the number of micro- and meso-pores.

### 2.2. Study of the Sorption Kinetics

**Figure 3:**
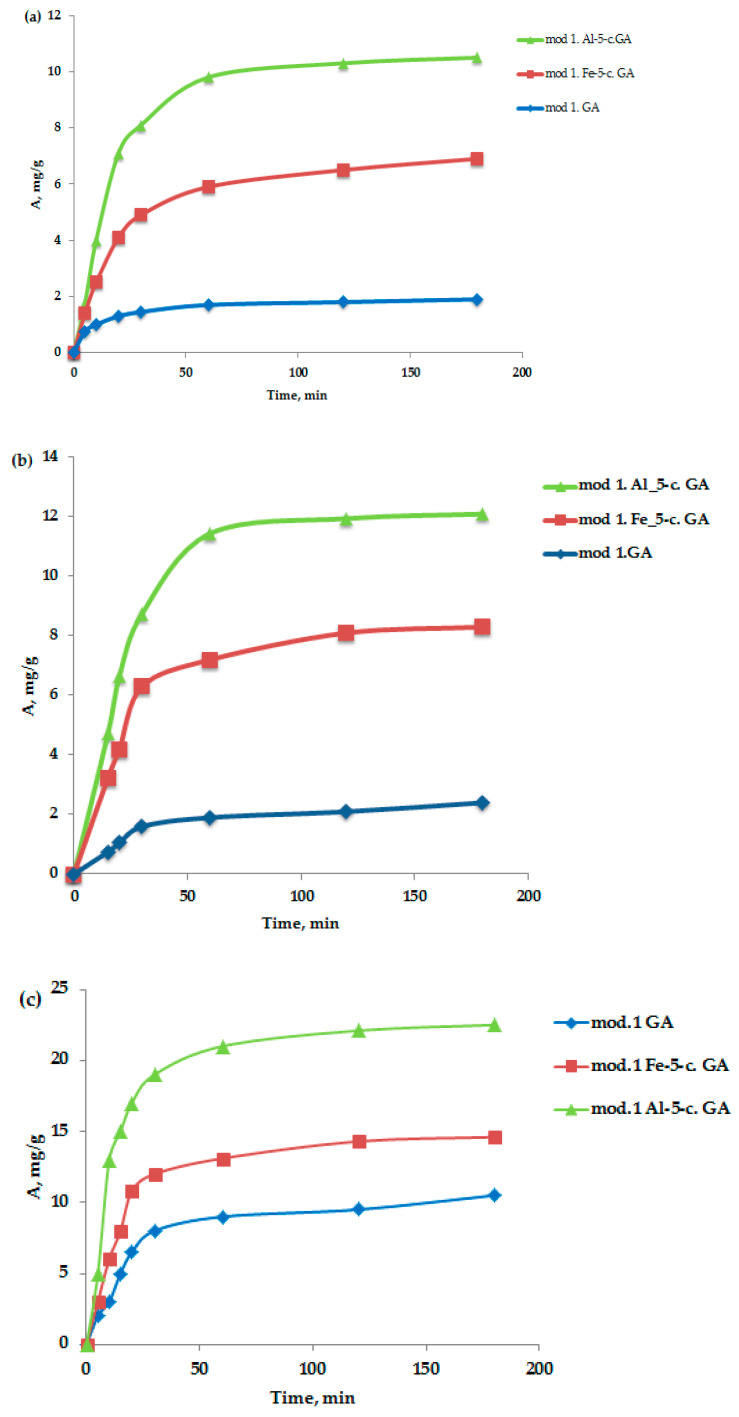
(**a**–**c**) Kinetic curves of the adsorption process of bichromate (**a**) and arsenate anions (**b**) and nickel cations (**c**) by the studied granular modified sorbents in a neutral medium.

An important characteristic in the study of the adsorption process is the kinetics of adsorption, which is necessary, firstly, to determine the time required to establish adsorption equilibrium when removing adsorption isotherms and, secondly, to establish the sorption mechanism. Oxygen-containing anions—bichromate and arsenate—were selected as test anions, as well as nickel (II) cations. The method of conducting the sorption experiment was as follows: samples of sorbents weighing 1–2 g were soaked in distilled water for 1 h; then the water was drained, and 100 mL of a model solution of arsenate and potassium bichromate of a certain concentration was added, mixing the adsorbent with the model solution. Samples of the solution for analysis were taken from the middle layers of the solution at 5, 10, 15, 20, 30, 60, 120, and 180 min. Quantitative analysis of the samples for the content of bichromate and arsenate anions, and nickel cations, was performed using an energy-dispersive X-ray fluorescence spectrometer (EDX-720) with calibration curves. Data on the kinetics of adsorption of bichromate and arsenate anions and nickel cations on the studied sorbents, obtained from bentonite sourced from the Pogodaevsky deposit, are shown in Figure 3a–c. The analysis of kinetic data on the sorption of bichromate and arsenate anions and nickel cations on the studied sorbents indicates that the saturation of sorbents with the studied sorbates under these conditions occurs within 2 h. Therefore, in the future, when removing the sorption isotherms, the time required to establish adsorption equilibrium will be 2 h.

### 2.3. Study of the Sorption Mechanism

#### 2.3.1. Isotherms of Sorption of Bichromate, Arsenate Anions and Nickel (II) Cations

Sorption of heavy metal ions is a complex process regulated by a number of factors [27,28]. Possible processes include chemisorption, complexation, and adsorption on the surface of the sorbent and within its pores, as well as ion exchange, microdeposition, and precipitation of heavy metal hydroxides.

The study of isotherms is the main method for examining the mechanisms of adsorption. Sorption isotherms display the equilibrium distribution of metal ions between the adsorbent and the liquid phase, depending on concentration [29]. The study of these isotherms helps draw conclusions about the nature of the sorbent surface and the interactions between the sorbate and sorbent. The Langmuir model was used to conduct the experiment.

The adsorption isotherms of bichromate and arsenate anions, as well as nickel (II) cations on the studied sorbents, were obtained as follows: similarly to the kinetic experiments, samples of the studied sorbents weighing 1–2 g were filled with distilled water for 1 h. The water was then drained, and the samples were filled with 100 mL of model solutions of bichromate and arsenate anions and nickel cations of various concentrations (100, 200, 300, 400, and 500 mg/L) and kept for 2 h until the equilibrium concentration in the solution was achieved. Samples of the solution for analysis were also taken from the middle layer. Quantitative analysis of the elemental composition of the sample was conducted using an energy-dispersive X-ray fluorescence spectrometer (EDX-720) and calibration curves.

In accordance with the average values of equilibrium concentrations (based on at least two parallel measurements), the adsorption value was calculated using Formula (1):

*A* = (*C_i_* − *C_e_*) × *V*/*m* (1)

*A*—adsorption capacity of the sorbent, mg/g;

*C_i_*—initial concentration of the studied ions in solution, mg/L;

*C_e_*—equilibrium concentration of the studied ions in solution, mg/L;

*V*—volume of the test solution, L;

*m*—the mass of the sorbent taken for analysis, g.

**Figure 4:**
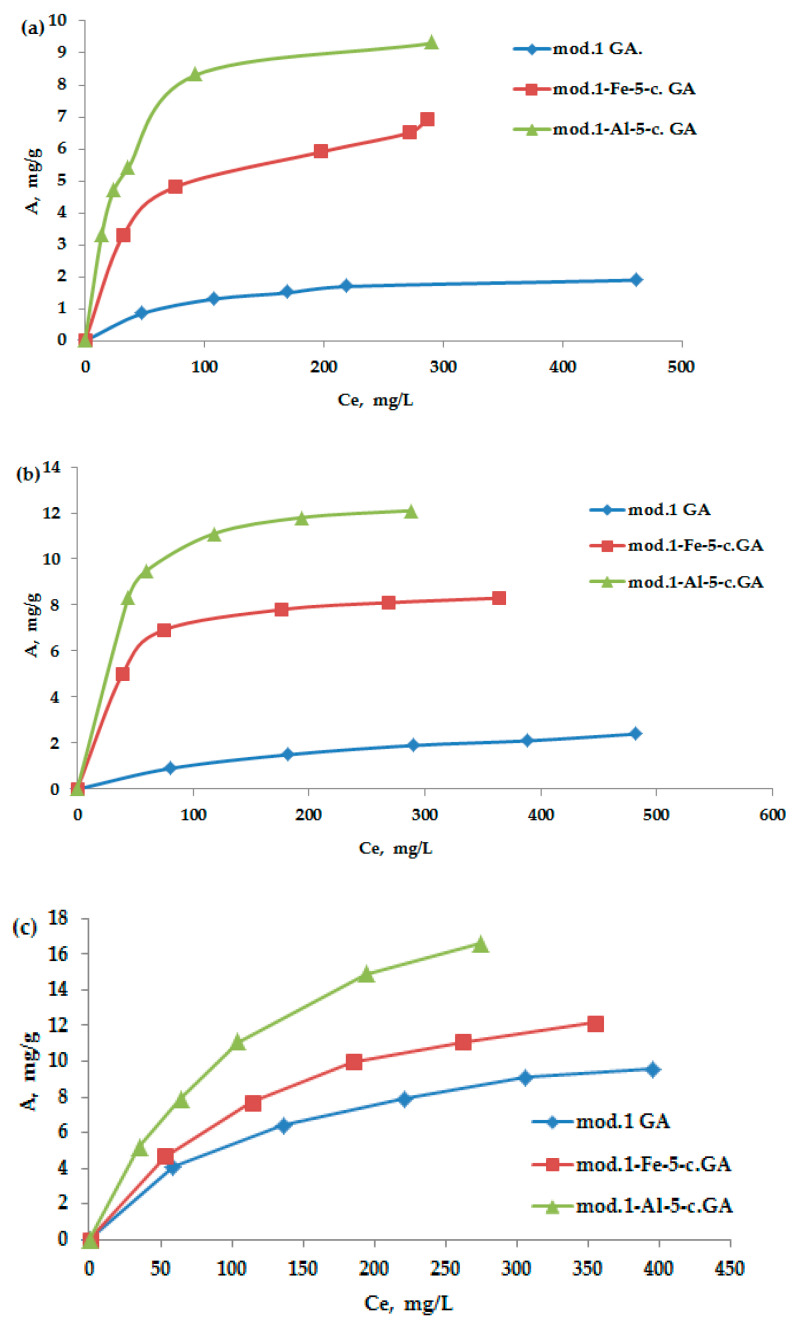
(**a**–**c**) Adsorption isotherms in a neutral medium of bichromate and arsenate ions and nickel cations on the obtained sorbents.

Figure 4a–c show the adsorption isotherms of bichromate and arsenate anions, as well as nickel cations, on the studied granular sorbents derived from natural bentonite from the Pogodayevo deposit. All obtained isotherms belong to the Langmuir type (*L-type*). The Langmuir adsorption isotherm equation, based on molecular kinetic theory and the monomolecular nature of the adsorption process, takes the form of Equation (2) in relation to solutions:

(2) $$A=\frac{A_{\infty }\cdot K\cdot C_{e}}{\left(1+K\cdot C_{e}\right)}$$

where *K* is the adsorption equilibrium constant characterizing the adsorption energy; *C_e_*—equilibrium concentration, mg/L; *A*_∞_—the maximum adsorption value, mg/g.

Adsorption isotherms are a useful tool for understanding the nature of sorbent surfaces. The Langmuir adsorption isotherm (2) is linearized in the coordinates 1/*A* = *f*(1/*C*), which allows for graphoanalytical determination of the values of the coefficients *K* and *A*_∞_. The obtained adsorption isotherms were processed in accordance with the Langmuir equation in inverse coordinates, as per Equation (3):

(3) $$\frac{1}{A}=\frac{1}{A_{\infty }}+\frac{1}{A_{\infty }K}\cdot \frac{1}{C}$$

**Figure 5:**
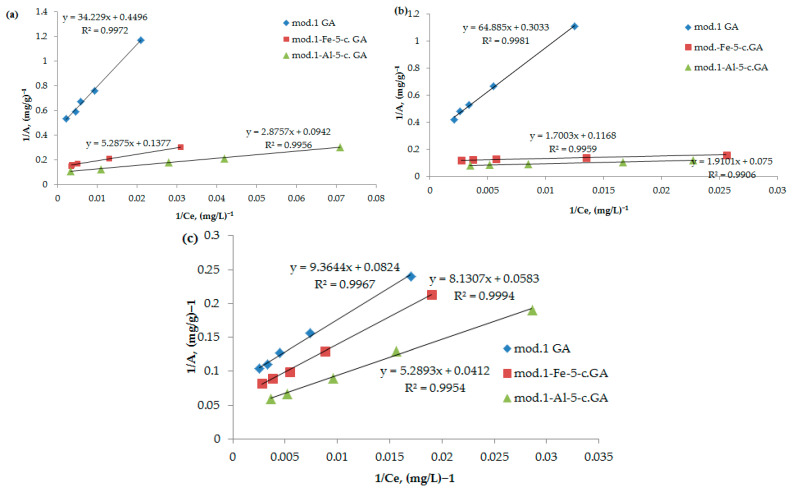
(**a**–**c**) Isotherms of adsorption of bichromate (**a**) and arsenate anions (**b**) and nickel cations (**c**) by the studied sorbents, represented in inverse coordinates in accordance with the Langmuir Equation (3).

Figure 5 illustrates the adsorption isotherms of the anions and cations examined, displayed in inverse coordinates based on the Langmuir Equation (3). By applying regression equations, we calculated the maximum adsorption values for the anions on both the initial bentonite and the modified sorbents, with the results shown in Table 4.

As follows from the obtained data, the modification of bentonite with iron (III) and aluminum compounds using the “co-precipitation” method leads to a significant increase in the maximum adsorption of the studied anions and cations. It should be noted that arsenate anions exhibit the highest sorption activity among the studied anions; the maximum adsorption value on the aluminum-modified sorbent is 13.3 mg/g. The adsorption value of nickel cations on the aluminum-modified sorbent reaches 24.3 mg/g.

#### 2.3.2. Kinetic Analysis of the Adsorption Processes of Bichromate and Arsenate Anions and Nickel Cations Occurring on Bentonites Modified by Polyhydroxocations of Metals

The rate of heterogeneous reaction, which includes the sorption of solutes on solid-phase sorbents, is influenced by numerous factors. Among these factors are the characteristics of both the sorbent material and the dissolved substances being absorbed [30]. The absorption process proceeds in several stages, the first of which depends on the diffusion coefficient of the solute in the surrounding solution. This is followed by a phase in which it is necessary to overcome the boundaries where the surface of the sorbent granules is covered with a thin liquid film separating them from the sorbent material. Next, there is a step involving the dispersion of sorbent molecules in the solid phase, influenced by various factors such as the size, charge, and hydration of the solute molecule, as well as the characteristics of the sorbent material, including type, charge density, pore size, and moisture content. Finally, there is a stage involving ion exchange. The complex, multi-stage nature of the sorption process makes it difficult to comprehensively consider all stages simultaneously, which leads to the use of kinetic models to determine the limiting stage of the process. Mathematical models, including external (4) and internal diffusion (5), pseudo-first-order (6), and pseudo-second-order reactions (7), are used to determine the limiting stage of sorption kinetics:

*F* = *q_t_*/*q_e_* (4)

*q_t_* = *K_p_t*^0.5^ + *C* (5)

ln(*q_e_* − *q_t_*) = ln*q_e_* − *k*_1_*t* (6)

*t*/*q_t_* = 1/*k*_2_*q_e_*^2^ + *t*/*q_e_* (7)

where *q_t_*—the adsorbed amount at time *t*, mg·g^−1^;

*q_e_*—the adsorbed amount in equilibrium, mg·g^−1^;

*k*_1_—the pseudo–first-order adsorption rate constant, min^−1^;

*k*_2_—the rate of adsorption constant of the pseudo-second-order, g (mg^−1^·min^−1^);

*K_p_*—the rate constant of intraparticle diffusion, mg·g^−1^·min^−0.5^).

The study of sorption kinetics allows us to identify factors that affect the dynamics and limit the speed of the process. The sorption rate is an important characteristic that determines whether the substance under study can be used as a sorbent. Typically, the rate of interaction between the sorbate and the sorbent is high in the first minutes of contact and then stabilizes at a steady level. To shorten the sorption cycle time in a technological or laboratory process, it is preferable to achieve sorption equilibrium as quickly as possible.

**Figure 6:**
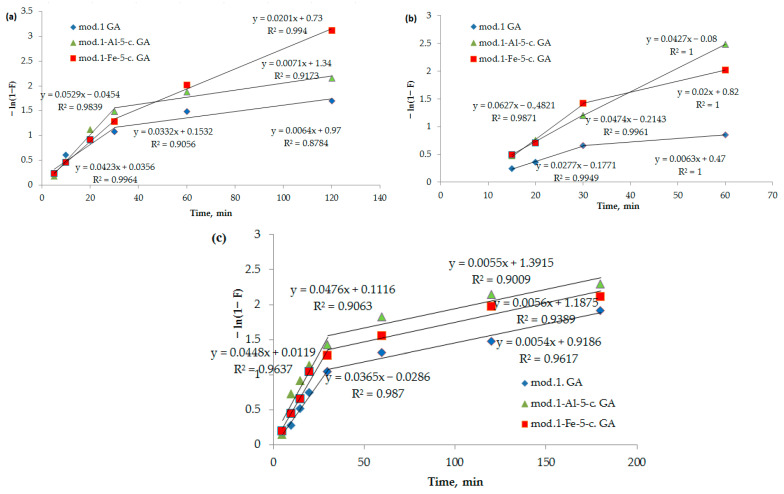
(**a**–**c**) Dependence of −ln(1 − *F*) on *t* (spring diffusion model) during adsorption of bichromate and arsenate anions and nickel cations by bentonite and modified iron (III) and aluminum polyhydroxocations by bentonite-based sorbents: (**a**) bichromate anion; (**b**) arsenate anion; (**c**) nickel cations.

It can be seen from Figure 6 that the kinetic adsorption profiles of bichromate and arsenate anions and nickel cations by the studied modified sorbents initially demonstrate linear behavior in the coordinates −ln(1 − *F*) as a function of time (*t*). This observation suggests that the sorption mechanism of these sorbents at the beginning is mainly determined by external diffusion. However, as the process progresses, the linearity of the curve decreases, indicating an increase in the intra-diffusion component. Consequently, the development of the process occurs due to a combination of diffusion mechanisms, including diffusion in the solution film and diffusion inside the sorbent grains.

**Figure 7:**
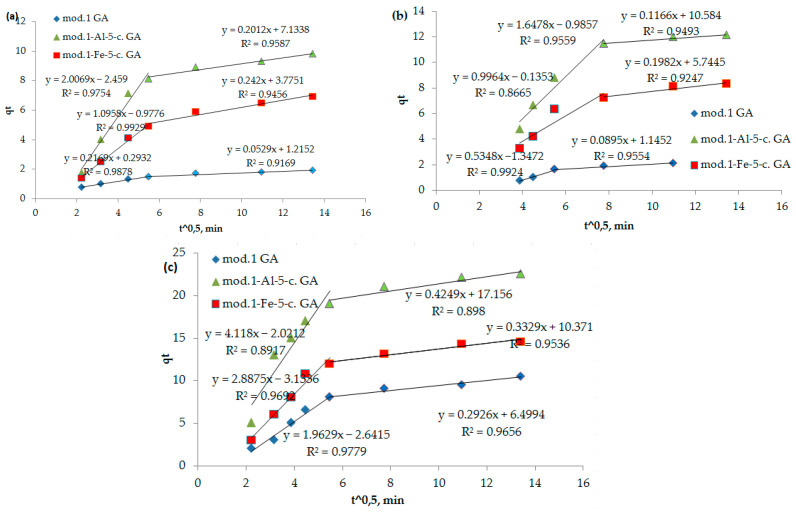
(**a**–**c**) Dependence of *q_t_* on *t*^0.5^ (internal diffusion model) during adsorption of bichromate and arsenate anions and nickel cations by bentonite and modified iron (III) and aluminum polyhydroxocations by bentonite-based sorbents: (**a**) bichromate anion; (**b**) arsenate anion; (**c**) nickel cation.

The proof that the stage limiting the sorption process is internal diffusion is the observation of a linear dependence of *q_t_* on *t*^0.5^ (Figure 7). The number of anions that were sorbed over time in a diffusion-controlled process can be mathematically described by Equation (5) [31].

As can be seen from Figure 7, these dependencies are multilinear and do not intersect with the origin. It follows from the above that in the initial period, the sorption process is controlled by an external diffusion factor, specifically the diffusion of the sorbate from the solution into a liquid film on the surface of the sorbent. Subsequently, the sorption process begins to proceed in a mixed diffusion mode, where it is limited by both the external diffusion factor and the internal diffusion factor (the diffusion of the sorbate to the active centers through a system of pores and capillaries). The segment cut off by the continuation of this straight line reflects the process of ion exchange occurring between the functional groups of the sorption materials (bentonites), bichromate and arsenate anions, and nickel cations.

The parameters of the adsorption of bichromate and arsenate anions and nickel cations by modified sorbents, obtained using kinetic diffusion models, are presented in Table 5.

**Figure 8:**
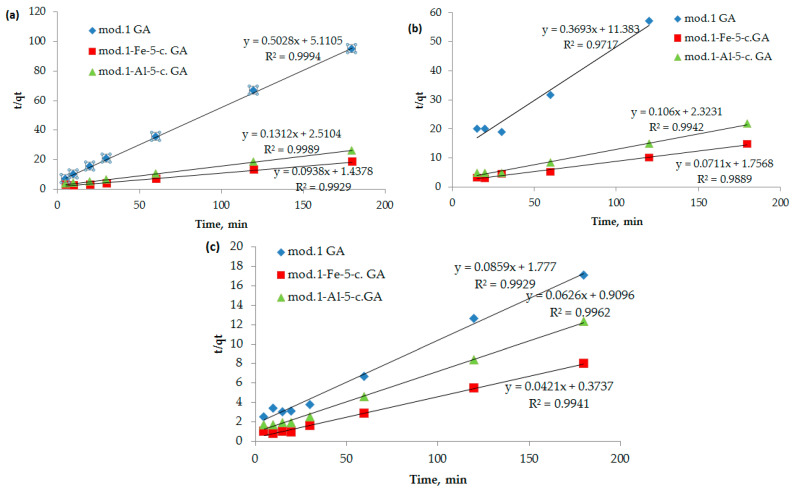
(**a**–**c**) Dependence of *t*/*q_t_* on *t* (a pseudo-second-order kinetic model) during the adsorption of bichromate and arsenate anions and nickel cations by bentonite and modified iron (III) and aluminum polyhydroxocations using bentonite-based sorbents: (**a**) bichromate anions, (**b**) arsenate anions, (**c**) nickel cations.

To clarify the contribution of the chemical stage to the rate of adsorption of bichromate and arsenate anions, and nickel cations by the studied sorbents, kinetic curves (Figure 8) were processed using equations of chemical kinetics (models: pseudo-first (6) and pseudo-second (7) orders).

The analysis of kinetic information obtained during the study of the adsorption processes of the studied anions and cations with granulated modified sorbents based on bentonite showed that the pseudo-first-order kinetic model (dependence of ln(*q_e_* − *q_t_*) on *t*) does not fit well (correlation coefficient is lower than 0.7). At the same time, the pseudo-second-order kinetic model (Equation (7)) fits satisfactorily, showing a linear dependence of *t*/*q_t_* on *t* with a correlation coefficient of 0.98 and higher (Figure 8, Table 6).

It follows from the data presented in Table 6 that the adsorption of bichromate and arsenate anions, as well as nickel cations, by modified sorbents based on natural bentonite is satisfactorily described by a kinetic model of pseudo-second order. The high correlation coefficient (R^2^ > 0.98) indicates a significant contribution of sorbate–sorbate interactions to the overall mechanism of adsorption [32]. This model allows us to describe the process from the point of view of chemical kinetics, demonstrating compliance with experimental data.

At the same time, the results of kinetic analysis show that the adsorption process has a two-stage nature [33]. At the first stage, external diffusion prevails, associated with the transport of sorbate through the liquid film to the sorbent surface. At the second stage, the process is limited by internal diffusion, in which sorbate molecules penetrate the porous structure of the sorbent and interact with the active centers. This combination of external and internal diffusion is consistent with the obtained kinetic curves and reflects the complex nature of the adsorption mechanism [34].

The pseudo-second-order kinetic model used to describe the process is also related to sorbate–sorbent interactions, which confirms its physicochemical validity. These interactions are caused by the structural features of the modified sorbents, including an increase in the number of active centers and a change in their energy of interaction with sorbates.

The kinetic curves shown in Figure 8, as well as the data on the maximum adsorption of the anions and cations under study (Figure 4 and Figure 5, Table 4), demonstrate a high degree of consistency. This indicates that the kinetic model of pseudo-second order correctly describes the adsorption process and also reflects the basic patterns of interaction of sorbates with modified sorbents [35].

Given the heterogeneous nature of adsorption processes, the properties of the sorbent surface, including its structure, chemical composition of the surface layer, and porosity, play a key role. Modification of sorbents with polyoxycomplexes of aluminum and iron leads to the creation of additional active centers, which increases the sorption capacity. Thus, the proposed models make it possible to explain the observed phenomena and mechanisms that determine the efficiency of adsorption.

## 3. Properties of the Obtained Granular Sorbents

### 3.1. Materials and Methods

Bentonite clay was obtained from the Pogodayevo deposit (West Kazakhstan region, Republic of Kazakhstan) and purified by precipitation combined with ultrasound treatment and centrifugation. Aluminum chloride (AlCl_3_·6H_2_O, 97%), iron chloride (FeCl_3_·6H_2_O, 97%), silver nitrate (AgNO_3_, 98%), and sodium hydroxide (NaOH, 98%) were sourced from Merck. Potassium arsenate and potassium bichromate (K_3_AsO_4_, K_2_Cr_2_O_7_, 98%, NiCl_2_, 98%) were used as model compounds.

Quantitative elemental composition analysis was conducted using an energy-dispersive X-ray fluorescence spectrometer EDX-720 (SHIMADZU, Kyoto, Japan) following the methodology of fundamental parameters. The porous structure of the specimens was assessed via low-temperature nitrogen adsorption using a high-speed gas sorption analyzer (Quantachrome NOVA, Anton Paar, Torrance, CA, USA). The specific surface areas of the solid samples were calculated using the Brunauer–Emmett–Teller method. The pore volume and size distribution were determined using the Barrett–Joyner–Halenda method. Initial data for calculations employing the BJH method were derived from the desorption or adsorption branch of the isotherm within the pressure range of 0.967 to 0.4 P/Po. X-ray phase analysis was performed using an X-ray diffractometer DRON-8T (Saint Petersburg, Russia). The powder was ground in an agate mortar and placed in a quartz cuvette 2 mm deep. CuKα radiation, a Goebel parabolic mirror (AXO Dresden GmbH, Dresden, Germany), and a position-sensitive Mythen 2R1D detector with 640 channels (Dectris, Baden, Switzerland) with a discretization of 2θ = 0.0144° were used to register diffractograms. The geometry of the focal beam included axial slits of 12 mm and equatorial slits of 0.25 mm. The registration was carried out by rotating the cuvette at 0.3 rpm in the range of angles from 5 to 80° with a step of 0.1° for the central channel of the detector, and an exposure time at each point of 2 s. The ability of the studied samples to absorb salt anions was assessed by constructing sorption isotherms using varying concentrations under statistical conditions. The model solution consisted of K_3_AsO_4_, K_2_Cr_2_O_7_, and NiCl_2_ salt solutions. Arsenic and chromium (III) anions and Ni cations were analyzed using atomic absorption spectroscopy (AAS, SHIMADZU-6800).

### 3.2. Preparation of Fe-Bentonite and Al-Bentonite

Modification of bentonites was carried out by the method of “co-precipitation” (intercalation or pillarization) [18]. FeCl_3_ and AlCl_3_ salts were added to the aqueous suspension of bentonite (the ratio of the solid to the liquid phase is 1:10 and the pH of the aqueous extract of the suspension is 8). The concentration of iron (aluminum) in the bentonite was 5 mmol Me^3+^/g. The suspension was then treated with ultrasound at a frequency of 22 Hz for 3 min. Ultrasonic treatment leads to the activation of the surface of the reagents. Next, a 0.5 M solution of sodium hydroxide ([OH^−^]/[Me^3+^] = 2.23) was subjected to aging at room temperature for one day. After 24 h, the resulting modified bentonite was separated from the liquid phase on a Buchner funnel using a vacuum pump, washed with water until a negative reaction to chloride and/or sulfate ions, and dried at 80 °C. Then, some samples were granulated by extrusion of clay “dough” or vortex rolling and subjected to heat treatment at 550 °C for one hour.

### 3.3. Obtaining Granular Sorbent Samples

Granular sorbents were used to increase the chemical and mechanical resistance of sorbents. The granulation of sorbents was carried out by the method of vortex rolling of bentonite granules in a high-speed rotating drum. The preliminary preparation of the raw materials included sieving them through a sieve with a mesh size of 0.5 mm. Large particles were rejected, and the target fraction was dried at a temperature of 40–60 °C with intensive ventilation in a thermal chamber, with periodic stirring throughout the day.

Next, the bentonite powder in a rotating drum is moistened with distilled water at a predetermined feed rate. Absorbing the liquid phase, the bentonite particles, under the action of centrifugal forces, fall onto the inner surface of the cylinder and roll into regular spherical granules. The granules were then passed through sieves and subjected to heat treatment at 550 °C. The granules were heat-treated in a rotating muffle furnace, with the chamber set at 25 rpm.

The optimal heat treatment temperature was determined as the minimum of the temperature range, with a holding time (with rotation) of the granulate of 2 h, ensuring compliance with the requirements of GOST R 51641-2000 [36] regarding the chemical and mechanical resistance of granules. The experimentally established optimal heat treatment temperature is 550 °C. The specific surface area of the granules was 55–65 m^2^/g. At a higher heat treatment temperature, the specific surface area can be reduced by 25%. At the same time, the adsorption activity of the sorbents also decreases.

**Figure 9:**
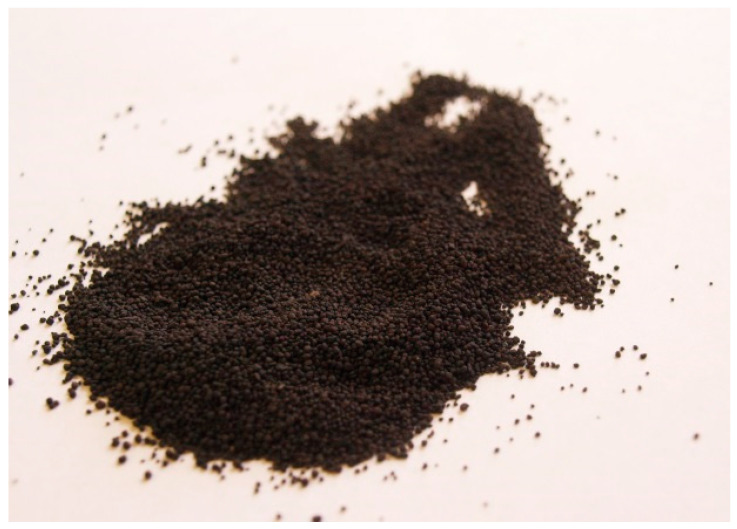
Image of the sorbent granulated using the vortex rolling method.

Figure 9 shows an image of the sorbent granulated by the vortex rolling method. It follows from the presented figure that the granulation method used allows for the formation of almost spherical, homogeneous granules with a size of 1–4 mm.

### 3.4. Assessment of Chemical and Mechanical Resistance of Granular Sorbents

The determination of the mechanical and chemical resistance of the granules of the obtained compounds was carried out according to GOST R 51641-2000 [36]. To determine the chemical resistance of granular sorbents, chemical analysis of neutral, alkaline, and acid extracts was performed after holding samples for one day in solutions of hydrochloric acid, sodium hydroxide, sodium chloride, calcium hypochlorite, and distilled water under static conditions. Chemical resistance was determined based on the results of repeated tests. The obtained solutions were then analyzed for permanganate oxidability, as well as the content of silicic acid, dry residue, aluminum, and iron. The chemical resistance in each model solution must meet the requirements of GOST.

The mechanical resistance of the material is characterized by its shreddability and abrasion resistance. After determining the chemical resistance, the samples of the material were thoroughly washed with distilled water, dried, and separately sieved on sieves № 2.0 and № 0.5. If the granulate passed through sieve № 2 and remained on sieve № 0.5, it was placed in flasks with distilled water. The flasks were then placed in the AWU-6s shaking apparatus, which provided 120 shakes per minute for 24 h. Afterward, the samples with the granulate were dried and sequentially dispersed on sieves № 0.25 and № 0.5. If the mass of the material (in grams) passed through sieve № 0.5 but remained on sieve № 0.25, this indicator characterized its shreddability and was expressed as a volume fraction of the total weight of the sample. The mass of the material (in grams) that passed through sieve № 0.25 characterized its abrasion and was also expressed as a volume fraction of the total weight of the sample material. The material passed mechanical tests if its abrasion resistance was not more than 0.5% and its shreddability was not more than 4%.

The results of repeated tests on the chemical resistance of sorbent granules (Table 7) correspond to the values established in GOST R 51641-2000 “Granular filtering materials” [36].

Data on the mechanical strength of the granular sorbent obtained by the vortex rolling method are shown in Table 8.

As a result of the conducted research, it was found that the shreddability of the obtained granular sorbents is 1.7%, and the abrasion is 0.2%, which is significantly lower than the GOST values. Therefore, by the method of vortex rolling followed by firing at a temperature of 550 °C, it is possible to obtain bentonite-based sorbent granules that meet the requirements of GOST.

### 3.5. Determination of Bulk Density of Granular Sorbents

For sorbents with a pronounced porous granule structure, one of the main physical characteristics is bulk density. The density of granules of aluminosilicate sorbents, due to their porous structure, has a somewhat conditional character, although this gravitational characteristic of sorbents is one of the main ones in the calculation of sorption filters. It would be more correct to call this physical characteristic of sorbents volumetric density, i.e., the density of the solid substance of the pellet in the volume occupied by the pellet, minus the volume of pores of this pellet filled with water.

The bulk density was determined in the following way. The sorbent weighing 500 g was placed in a drying cabinet and kept at a temperature of 60 °C for two hours. A weight of 400 g was taken from the dried mass of the sorbent. This suspension was placed into a measuring cylinder with a capacity of 500 mL, and the volume of the sorbent, V1, was measured. Then, by lightly tapping the bottom of the cylinder on the table, the sorbent was compacted to a constant sorbent level, and the volume of the sorbent in the cylinder, V2, was measured again.

The bulk density of the sorbent in g/cm^3^ was calculated using Formula (9):

(8) $$\gamma_{1}=\frac{P}{V_{1}};\;\gamma_{2}=\frac{P}{V_{2}}$$

where *P* is the suspension of the sorbent, g;

*γ*_1_, *γ*_2_ are the bulk density of the sorbent before and after compaction, g/cm^3^.

As the sorbent under study, a sample of mod. 1 GA was taken—the initial bentonite (Pogodayevo), granulated by vortex rolling and annealed at 550 °C in an inert argon atmosphere. The results of the bulk density measurements for the sorbent are presented in Table 9.

The bulk density for all samples does not exceed the maximum permissible values (*γ* = 1.4 g/cm^3^) of TC 2163-001-01115840-94.

## 4. Conclusions

1. A method for producing effective granular sorbents based on bentonite modified with polyhydroxocations of aluminum and iron (III) by the “co-precipitation” method has been developed; their physicochemical and adsorption properties with respect to nickel (II) cations and the oxygen-containing anions arsenate and bichromate have been studied.
2. X-ray phase analysis has established that the additional introduction of aluminum and iron (III) polyhydorxocations into bentonite by the “co-precipitation” method does not lead to a change in the mineral and phase composition of bentonite (in all considered cases, the following minerals are observed: montmorillonite, α—cristobalite, plagioclase). Annealing of the studied sorbent samples at 550 °C in an argon atmosphere leads to changes in the mineral composition of the sorbents: instead of montmorillonite, the mineral illite appears.
3. It was found that modification of bentonite with aluminum and iron (III) polyhydroxocations leads to an increase in the total specific surface area (up to 54–65 m^2^/g). High temperature (550 °C) leads to a slight decrease in the specific surface area of the sorbents under study. It is shown that modified bentonite-based sorbents are micro-porous materials that are thermally stable at temperatures up to 550 °C: most of the pores of all modified samples are pores with a size of 1.5–8.0 nm.
4. The process of adsorption by modified sorbents based on bentonite of bichromate and arsenate anions and nickel (II) cations has been studied. It has been established that the obtained adsorption isotherms of the studied anions and cations belong to Langmuir-type isotherms. Modification of bentonite with polyhydroxocations of aluminum and iron (III) leads to a significant increase in the adsorption characteristics of the studied sorbents in relation to the studied anions and salt cations, which is due to an increase in the number of anion exchange centers on the modified sorbents.
5. Kinetic analysis showed that at the initial stage the sorption process is controlled by an external diffusion factor, i.e., the diffusion of the sorbent from the solution to the liquid film on the surface of the sorbent. Then the sorption process begins to proceed in a mixed diffusion mode, when both the external diffusion factor and the intra-diffusion factor are activated (diffusion of the sorbent to the active centers through the system of pores and capillaries).
6. To clarify the contribution of the chemical stage to the rate of adsorption of bichromate and arsenate anions and nickel cations by the studied sorbents, kinetic curves were processed using equations of chemical kinetics (models: pseudo-first and pseudo-second orders). As a result, it was found that the adsorption of the studied anions and cations by modified sorbents based on natural bentonite is best described by a pseudo-second-order kinetic model, as evidenced by the correlation coefficient (R^2^ > 0.98), which allows us to draw conclusions about the contribution of sorbate–sorbate interactions during sorption of anions to the overall rate of the process.
7. Based on the fact that there are structural disorders in the porous system of the studied sorbents and their surface can be considered as a heterogeneous system, and considering that heterogeneous processes occur on the surface of the sorbent, it is natural that all surface properties (structure, chemical composition of the surface layer, etc.) play an important role in the process of anion adsorption.
8. It is shown that the use of natural bentonite for the development of technology for the production of granular sorbents based on it has an undeniable advantage: firstly, in terms of its chemical and structural properties, it is easily and effectively modified, and secondly, having astringent properties, granules are easily made on its basis, which, during high-temperature firing, they turn into ceramics. The result is a granular sorbent with high physical and mechanical properties. Since bentonite is an environmentally friendly product, the technology of recycling spent sorbents is also greatly simplified.

## Figures and Tables

**Table 2:** The main characteristics of the porous structure of the studied granular sorbents based on bentonite modified with polyhydroxocations of iron (III) and aluminum (III) by the “co-precipitation” method.

Sample	Specific Surface Area, m^2^/g	Pore Volum, cm^3^/g	Distribution of Pores by Radius, %			
			1.5–2.0 nm	2.0–4.0 nm	4.0–8.0 nm	More than 8.0 nm
mod. 1 GA	26	0.064	5	13	23	59
mod. 1_Al_5-c GA	65	0.073	16	35	14	35
mod. 1_Fe_5-c GA	54	0.068	11	24	20	45

**Table 3:** Particle size distribution (%) for the studied powdered sorbent samples obtained by the “co-precipitation” method.

Sample	mod. 1 GA	mod. 1_Fe_5-c GA	mod. 1_Al_5-c GA
Particle diameter, microns			
0.5–5	-	0.5 ± 0.05	1.0 ± 0.1
5–10	1.0 ± 0.1	1.5 ± 0.1	-
10–50	11 ± 1	14 ± 1	17 ± 1
50–100	29 ± 1	36 ± 2	29 ± 1
100–200	46 ± 2	34 ± 2	42 ± 2
200–500	13 ± 1	14 ± 1	11 ± 1

**Table 4:** Values of the maximum adsorption capacity of the studied anions and cations for the studied bentonite-based sorbents.

Sample	*A*∞, mg/g		
	Arsenate Anions	Bichromate Anions	Nickel Cations
mod. 1 GA	3.3	2.2	12.1
mod. 1 Al_5-c GA	13.3	10.6	24.3
mod. 1 Fe_5-c GA	8.6	7.3	17.1

**Table 5:** Results of processing experimental kinetic adsorption curves of bichromate and arsenate anions and nickel cations with modified bentonite-based sorbents using diffusion models.

Sample	The Model of External Diffusion	Internal Diffusion Model		
	R^2^	*K* *_p_*	C	R^2^
arsenate anions				
mod. 1 GA	0.99	0.089	1.15	0.96
mod. 1_Al_5-c GA	0.99	0.198	5.70	0.92
mod. 1_Fe_5-c GA	0.99	0.117	10.6	0.95
bichromate anions				
mod. 1 GA	0.91	0.053	1.22	0.92
mod. 1_Al_5-c GA	0.99	0.242	3.77	0.95
mod. 1_Fe_5-c GA	0.98	0.241	7.13	0.96
nickel cations				
mod. 1 GA	0.99	0.293	6.50	0.97
mod. 1_Al_5-c GA	0.96	0.333	10.4	0.95
mod. 1_Fe_5-c GA	0.91	0.425	17.6	0.90

**Table 6:** Results of processing experimental kinetic curves of the adsorption of bichromate and arsenate anions and nickel cations with granular sorbents based on modified bentonite using a pseudo-second-order kinetic model.

Sample	Pseudo-Second Order		
	*q_e_*	*k*_2_	R^2^
	arsenate anions		
mod. 1 GA	2.7	0.0120	0.97
mod. 1_Al_5-c GA	9.4	0.0049	0.99
mod. 1_Fe_5-c GA	14.1	0.0029	0.99
	bichromate anions		
mod. 1 GA	2.2	0.4498	0.99
mod. 1_Al_5-c GA	11.6	0.0859	0.99
mod. 1_Fe_5-c GA	8.6	0.1158	0.99
	nickel cations		
mod. 1 GA	11.6	0.0042	0.99
mod. 1_Al_5-c GA	15.9	0.0043	0.99
mod. 1_Fe_5-c GA	23.8	0.0047	0.99

**Table 7:** Indicators of chemical resistance of granules of the studied granulated sorbents based on bentonite, obtained by vortex rolling and annealed at 550 °C.

The Name of the Indicator	Experimental Values
Increase in oxidizability, mg/dm^3^	<10
Increase in the mass concentration of silicic acid in terms of silicon, mg/dm^3^	<10
Increase in dry residue, mg/dm^3^	<20
Increase in the total mass concentration of aluminum and iron in terms of (II) oxides, mg/dm^3^	<2.0

**Table 8:** Indicators of the mechanical strength of the granular sorbent obtained by the vortex rolling method.

Indicator	Granulometric Composition, mm	Sorbent Sample	Experimental Data, %	GOST, No More, %
Shreddability	3.0–2.0	mod. 1 GA	1.5	4.0
		mod. 1_Al_5-c GA	1.6	
		mod. 1_Fe_5-c GA	1.8	
		av.	1.7	
Abradability	less than 2.0	mod. 1 GA	0.1	0.5
		mod. 1_Al_5-c GA	0.2	
		mod. 1_Fe_5-c GA	0.3	
		av.	0.2	

**Table 9:** Bulk density of the studied sorbents.

Sorbent Samples	Granule Size, *d*_av_, mm	Bulk Density, g/cm^3^	
		*γ*_1_	*γ*_2_
mod. 1 GA	2.0–3.0	0.95	1.10
mod. 1_Al_5-c GA		0.91	1.04
mod. 1_Fe_5-c GA		0.93	1.06

## Data Availability

Data are contained within the article.
